# A small molecule increases mycobacterial translation fidelity without a fitness cost by targeting ribosomal protein S5

**DOI:** 10.1128/mbio.02056-26

**Published:** 2026-10-02

**Authors:** Jie Wu, Swarnava Chaudhuri, Sarah Caldwell Feid, Hemant Joshi, Miaomiao Pan, Qingwen Kawaji, Gang Liu, Babak Javid

**Affiliations:** 1 School of Pharmaceutical Sciences, Tsinghua University 12442, Beijing, P. R. China; 2 School of Medicine, Tsinghua University 12442, Beijing, P. R. China; 3 University of California San Francisco 8785, San Francisco, California, USA; Massachusetts Institute of Technology, Cambridge, Massachusetts, USA

**Keywords:** mistranslation, translational fidelity, small molecule, antibiotic tolerance, medicinal chemistry, mycobacteria

## Abstract

A central dogma of molecular biology is the “speed-accuracy trade-off,” where ribosomes must slow down to ensure accurate protein synthesis. In mycobacteria, a high basal level of mistranslation at glutamine and asparagine codons, caused by an indirect tRNA aminoacylation pathway, promotes tolerance to the antibiotic rifampicin. While pharmacologically increasing translational fidelity is a promising strategy to combat antibiotic tolerance, the underlying mechanisms remain poorly understood. Here, we screened 9,000 synthetic compounds and identified benzo[d]isoxazole-4,7-diones as a novel chemical class that reduces mycobacterial mistranslation. Medicinal chemistry optimization yielded a lead compound, 9787, with superior potency in decreasing mistranslation and reversing rifampicin tolerance. Using competitive chemical proteomics, we identified the 30S ribosomal protein S5 (RpS5) as the specific cellular target. Remarkably, compound 9787 enhances translational fidelity at concentrations that do not measurably impact the overall rate of protein synthesis. Our findings challenge the universality of the speed-accuracy trade-off, demonstrating that fidelity can be improved independently of translation speed. This work reveals that the ribosomal small subunit is a druggable target for modulating translational quality control, and introduces a new strategy for combating antibiotic-tolerant bacteria without the associated fitness cost of slowed translation.

**IMPORTANCE:**

A fundamental principle in molecular biology holds that ribosomes face a trade-off between translation speed and accuracy: going faster means making more errors, while maintaining high fidelity requires slowing down. This study challenges that paradigm by identifying a small molecule that increases translational accuracy in mycobacteria without affecting the rate of protein synthesis. The compound targets ribosomal protein S5 and specifically reduces errors arising from physiologically mischarged tRNAs—a quality control problem distinct from the well-studied codon-anticodon mismatches. This form of mistranslation contributes to antibiotic tolerance in tuberculosis, making it a potential therapeutic target. Our findings reveal unexpected flexibility in how ribosomes maintain translation quality and suggest that pharmacologically increasing fidelity without the fitness cost of slowed protein synthesis may be an attractive strategy for combating antibiotic-tolerant bacteria.

## INTRODUCTION

Genetically susceptible bacteria may still withstand killing by antibiotics, a phenomenon known as antibiotic tolerance (1, 2). There are several potential mechanisms for tolerance, and these may reflect different physiological states of the bacteria (3–5). Non-replicating persisters are usually multi-drug tolerant and have a relatively, but not entirely, quiescent physiological state (5–8). We recently described a form of tolerance in mycobacteria to the first-line antibiotic rifampicin, which we termed RNA polymerase-specific phenotypic resistance (RSPR) (9–11). By contrast with non-replicating persisters, the antibiotic-tolerant bacteria in RSPR are able to grow in bulk-lethal concentrations of antibiotic that can kill the majority of bacilli in the culture (9). Importantly, RSPR was apparent in *Mycobacterium tuberculosis* freshly isolated from sputum from a small number of patients, suggesting that it may be a clinically relevant mode of antibiotic tolerance (10, 12).

**Fig 1:**
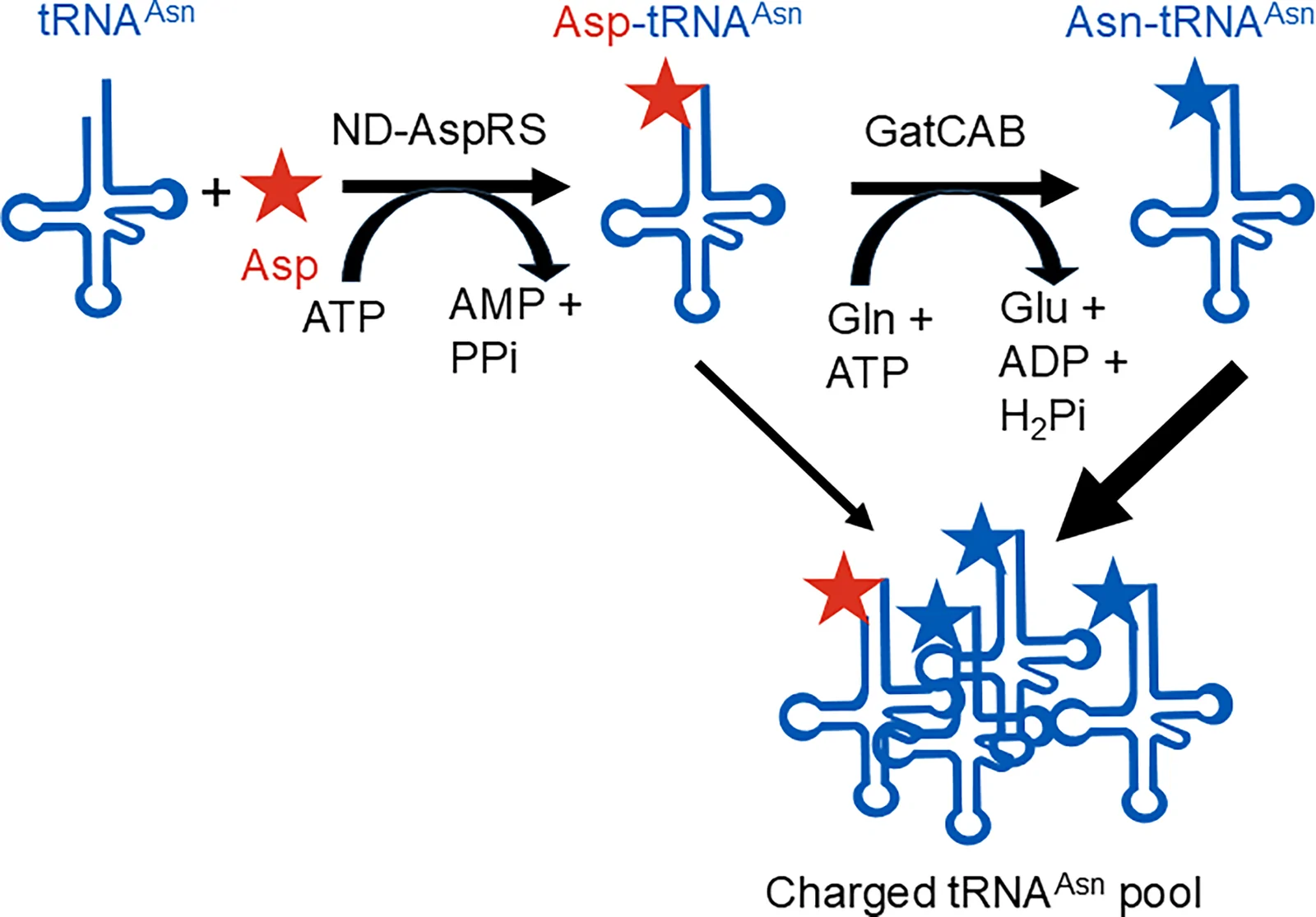
An error-prone two-step process is required for asparagine (and glutamine) tRNA aminoacylation in mycobacteria. The Asn tRNA pathway is illustrated for clarity. A non-discriminatory aspartyl-synthetase misacylates asparagine tRNA with Asp to Asp-tRNA^Asn^. The misacylated tRNA is recognized by the glutamine amidotransferase GatCAB, which amidates the misacylated tRNA using cytosolic glutamine as an amide donor. This results in a cognately charged Asn-tRNA^Asn^. The process for charging glutamine tRNAs is the same, using glutamate as the initial amino acid and a non-discriminatory glutamyl-synthetase. The relative inefficiency of GatCAB results in some mischarged tRNAs in the pool, which can proceed to partake in protein synthesis, resulting in misincorporation of Asp for Asn (and Glu for Gln).

We had identified at least two mechanisms that contribute to mycobacterial RSPR (10, 11). One of these mechanisms involved modulation of translational fidelity in the indirect tRNA aminoacylation pathway—or specific mycobacterial mistranslation (10, 13). Misincorporation of an aspartate for asparagine in a critical region of the rifampicin-binding domain of RNA polymerase (RNAP) allowed a fraction of synthesized RNAP to be inherently resistant to rifampicin-mediated inhibition, thereby permitting ongoing survival and growth in the presence of otherwise lethal concentrations of drug (9, 10). Unlike cytosolic eukaryotic gene translation, the vast majority of bacteria, except *Escherichia coli* and a few related proteobacteria, lack the specific aminoacyl-tRNA synthetases for either glutamine or asparagine, or both (14–18). All mycobacteria lack both glutaminyl- and asparaginyl-synthetases. Instead, they undergo a two-step pathway to specifically aminoacylate glutamine and asparagine tRNAs (Fig. 1). First, a non-discriminatory glutamyl- (or aspartyl-)synthetase physiologically misacylates the glutamine or asparagine tRNA, respectively, to Glu-tRNA^Gln^ and Asp-tRNA^Asn^. This misacylated complex is then specifically recognized by the essential glutamine amidotransferase GatCAB (10). Using cytosolic glutamine as an amide donor, GatCAB amidates the misacylated tRNAs to their cognate Gln-tRNA^Gln^ and Asn-tRNA^Asn^ respectively, thereby allowing preservation of the genetic code (Fig. 1). Therefore, mutations that alter GatCAB structure and stability can increase mistranslation in mycobacteria (10, 13). Moreover, we identified that naturally occurring mutations in *gatA* in clinical isolates of *Mycobacterium tuberculosis* both increased rates of specific mistranslation and tolerance to rifampicin (10). Furthermore, GatCAB could be limiting in wild-type mycobacteria, and sorting cells by decreased expression of wild-type *gatCAB* in an isogenic population identified subpopulations with both increased rates of mistranslation and antibiotic tolerance (10). We also showed that mistranslation of a specific, critical residue in the rifampicin-binding region of the cellular target of rifampicin, RNA polymerase (RNAP), contributed substantially to RSPR in wild-type mycobacteria (10). Together, these data compellingly suggested that specific mycobacterial mistranslation was an important contributor to RSPR.

Since mycobacterial mistranslation contributed to RSPR even in isogenic wild-type bacteria, we hypothesized that this pathway may be amenable to pharmacological intervention. We identified the natural product kasugamycin as a small molecule that could both decrease specific mycobacterial mistranslation and potentiate rifampicin both *in vitro* and in a murine model of tuberculosis (TB) (19). However, other than the naturally occurring peptide Edeine, which showed some activity in our assays (19), no other molecules capable of decreasing this specific form of bacterial mistranslation, which is mechanistically distinct from ribosomal decoding errors (10), have been identified. We decided to perform a pathway-specific whole-cell screen (20) that could identify synthetic small molecules that could target mycobacterial mistranslation. In this study, we utilized our mistranslation inhibition assay, which had earlier been used to identify kasugamycin’s ability to increase mycobacterial translational fidelity (19, 21), in a high-throughput format. Here, we identify analogs of benzo[d]isoxazole-4,7-diones that can decrease mistranslation rates and potentiate rifampicin *in vitro*. Furthermore, we identify the potential target of the most potent analog as the small ribosomal subunit protein RpS5. Importantly, the lead compound, 9787, increases translational fidelity at concentrations that do not impact the rate of protein synthesis, challenging the universality of the speed-accuracy trade-off and revealing previously unrecognized mechanisms of ribosomal quality control.

## RESULTS

### Development of a mistranslation pathway-specific phenotypic screen

**Fig 2:**
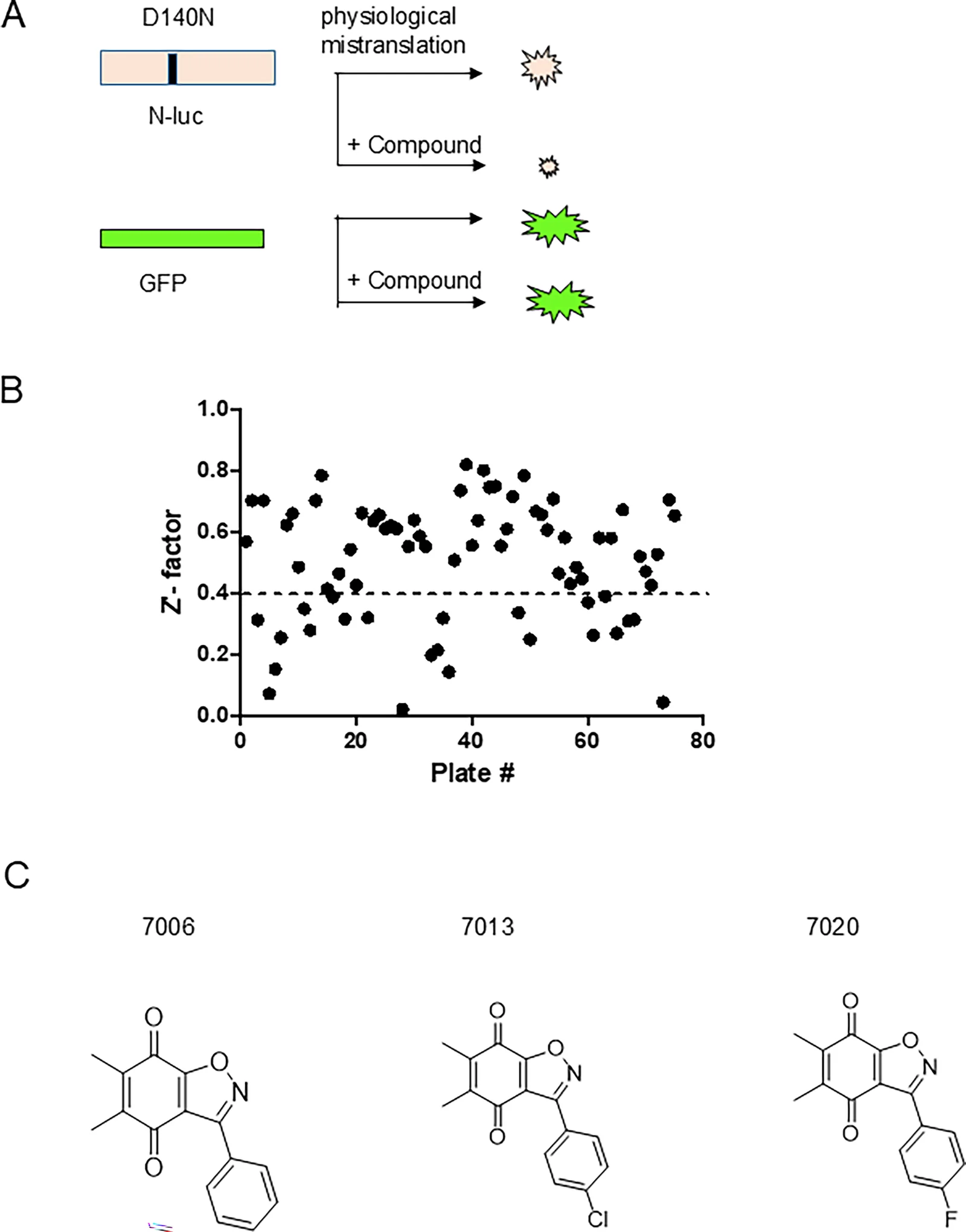
A phenotypic whole-cell screen identified benzo[d]isoxazole-4,7-dione analogs as modulators of mycobacterial mistranslation. (**A**) Schematic of the N-luc luciferase/GFP reporter system for screening of chemicals that decrease mistranslation in *Mycobacterium smegmatis*. (**B**) Screening of chemical library using the N-luc luciferase/GFP reporter for inhibitors of mycobacterial mistranslation (see Materials and Methods for details). The quality of the assay was assessed by the *Z*-factor, which was calculated by the formula *Z*-factor = $1-\frac{3\sigma_{p}+3\sigma_{n}}{\left|\mu_{n}-\mu_{p}\right|}$, where σ_p_, σ_n_, μ_p_, and μ_n_ are standard deviations (σ) and means (μ) of the positive (p) and negative (n) controls, respectively. Throughout the screening process, kasugamycin (Ksg) and dimethyl sulfoxide (DMSO) have been used as the positive and negative controls, respectively. (**C**) Structure of benzo[d]isoxazole**-**4,7-dione analogs identified in the preliminary screen (7006, 7013, and 7020).

We developed a reporter system in *M. smegmatis* to sensitively measure relative mistranslation of asparagine to aspartate—one of the two forms of mistranslation implicated in the indirect tRNA aminoacylation pathway (Fig. 2A) (19, 21). This whole-cell reporter system avoids a common pitfall of *in vitro* screens: such screens identify many hits that are ultimately inactive against whole cells due to the complex and thick mycobacterial cell wall and envelope (20). However, unlike a traditional whole-cell screen in which all hits that could kill bacteria are identified, leading to “re-discovery” of common anti-microbials or identification of non-specifically toxic molecules, the system retains the advantage of being “pathway specific.” Only hits that decrease specific mycobacterial mistranslation are identified.

The basis of the screen was as follows: Nluc luciferase was mutated at a critical aspartate residue (D140) to asparagine, which resulted in >99% loss of activity (19, 21). Physiological mycobacterial mistranslation of asparagine to aspartate resulted in a baseline detectable Nluc activity of ~1% of the wild-type enzyme (corrected for cell density and protein expression by normalizing to GFP fluorescence). If a small molecule *decreased* mistranslation rates, the corrected Nluc/GFP would be reduced below the baseline physiological rates (Fig. 2A). Hits were classified as those molecules that decreased mistranslation rates by >1 standard deviation compared with the negative control.

**Fig 3:**
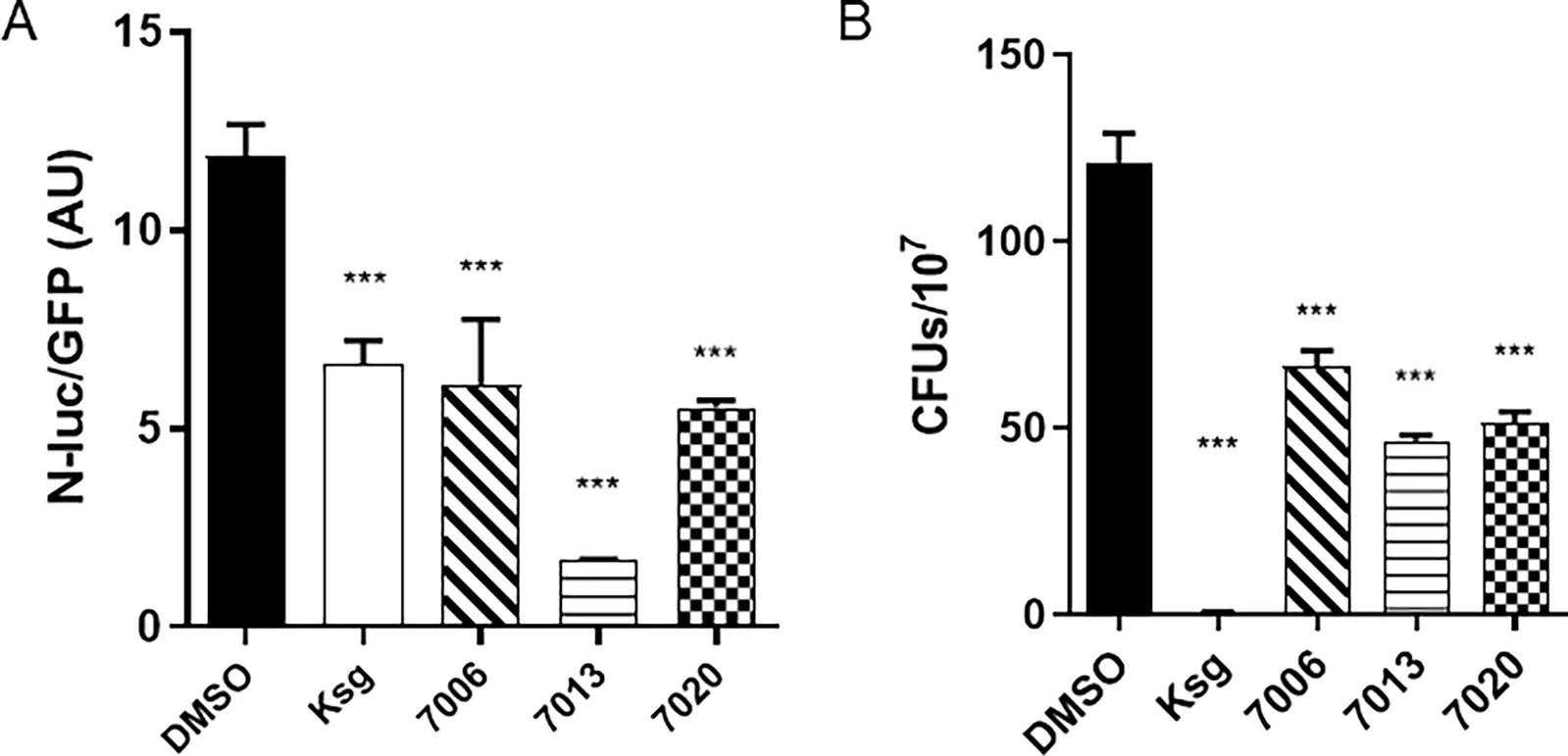
The benzo[d]isoxazole-4,7-dione analogs 7006, 7013, and 7020 decrease mycobacterial mistranslation and RSPR. (**A**) The compounds 7006, 7013, and 7020 (all at 50 μg/mL) significantly decrease mistranslation in *M. smegmatis* expressing the N-luc/GFP reporter system. (**B**) *M. smegmatis* was plated on rifampicin-containing medium (100 μg/mL) in the presence of 7006/7013/7020 (all at 50 μg/mL). All of the compounds could significantly enhance rifampicin-mediated mycobacterial killing. DMSO and kasugamycin (Ksg) were used as negative and positive controls, respectively, in the experiments. ***P < 0.001 by Student’s *t*-test.

We then used this mistranslation reporter strain to screen 9,000 synthetic heterocyclic small molecules in 96-well plate format (Fig. 2B). Screen performance was variable, due to the inherent noisy signal in measuring low rates of translational error, with 30% of plates yielding *Z*′ < 0.34 and excluded from analysis. From well-performing plates, we identified 45 hits. Of note, three were from the same chemical scaffold, benzo[d]isoxazole-4,7-diones (Fig. 2C). These three compounds, 7006, 7013, and 7020, were retested for their ability to reduce mycobacterial mistranslation and RSPR, confirming them as valid hits (Fig. 3A and B).

### Medicinal chemistry identifies potent analogs that increase specific translational fidelity

**Fig 4:**
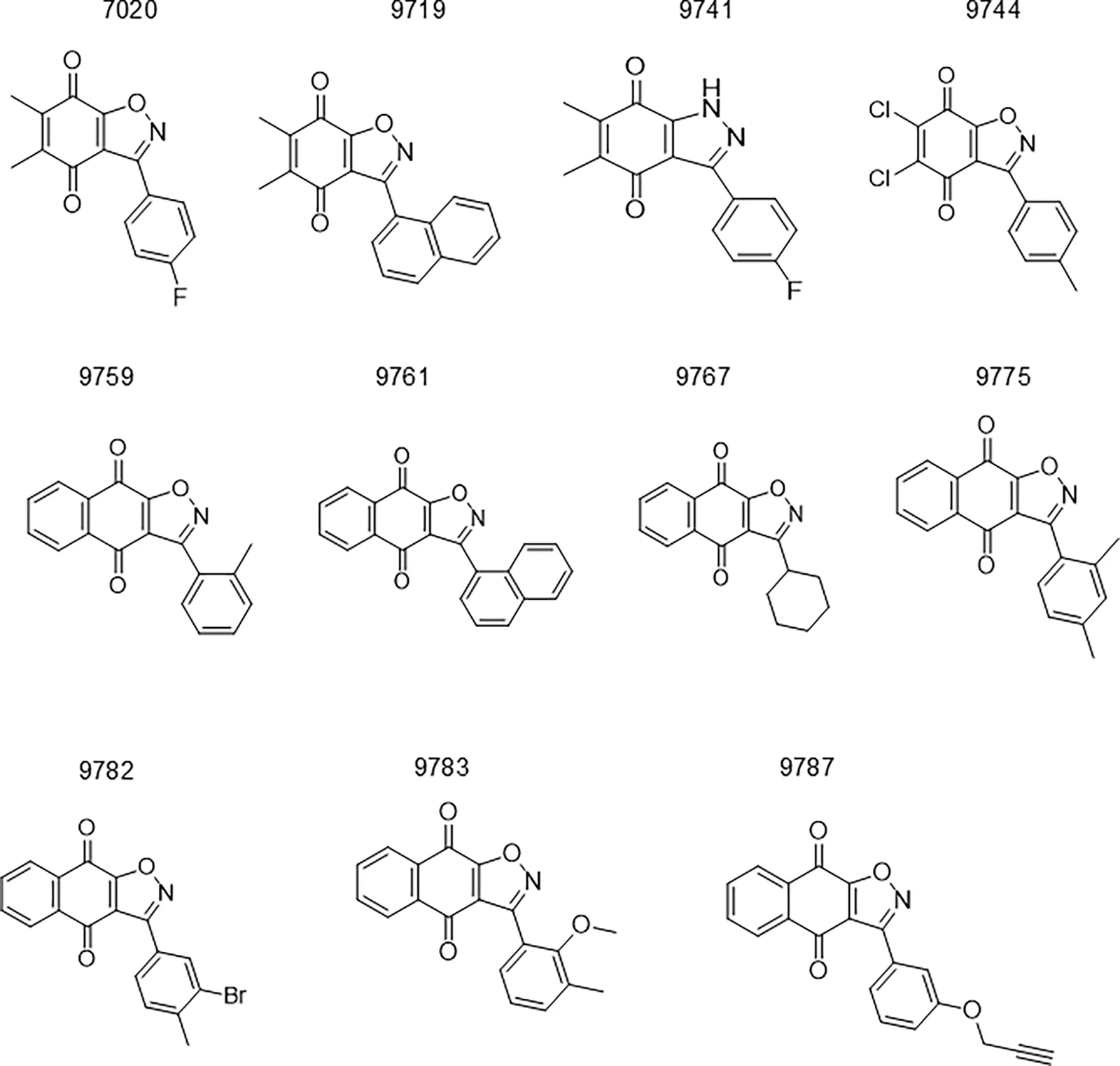
Structural analogs of 7020 that decrease mistranslation and RSPR in *M. smegmatis.* Several analogs of 7020 were synthesized, of which 10 compounds were found to be better than 7020 in inhibiting mycobacterial mistranslation and RSPR (Table 1). The structures of the analogs are shown here along with the parent compound 7020.

We prioritized 7020 for medicinal chemistry because it modulated translational fidelity without direct antimicrobial activity and was amenable to synthetic modification. This would allow us to deconvolute antimicrobial activity from anti-mistranslation activity. We synthesized 110 analogs of 7020, of which 10 compounds (Fig. 4) showed superior potency to the parent compound, 7020, both in terms of decreasing mistranslation and potentiating rifampicin in an assay used to measure RSPR (Table 1). The compounds also had minimal direct antimicrobial activity by themselves (Table 1), making them suitable for dissecting the contribution of targeting mycobacterial mistranslation on antibiotic tolerance without confounding issues due to inhibitory activity.

**TABLE 1:** Bioactivity of analogs of 7020 with superior potency^*a*^

Compound	Mistranslation inhibition (%)	RSPR inhibition (%)	Bacterial growth restriction in the absence of rifampicin (%)
7020	53.24	57.69	0
9719	83.21	61.23	0
9741	74.38	78.34	0
9744	86.95	87.32	1
9759	59.64	86.02	0
9767	64.77	81.43	1.2
9775	86.89	71.35	0
9782	83.73	86.78	0
9783	66.99	88.93	0
9787	75.51	98.14	1.15

^*a*^ Compounds with activity in the mistranslation assay (Fig. 3) were tested for activity on RSPR. Results are means of biological triplicates.

**Fig 5:**
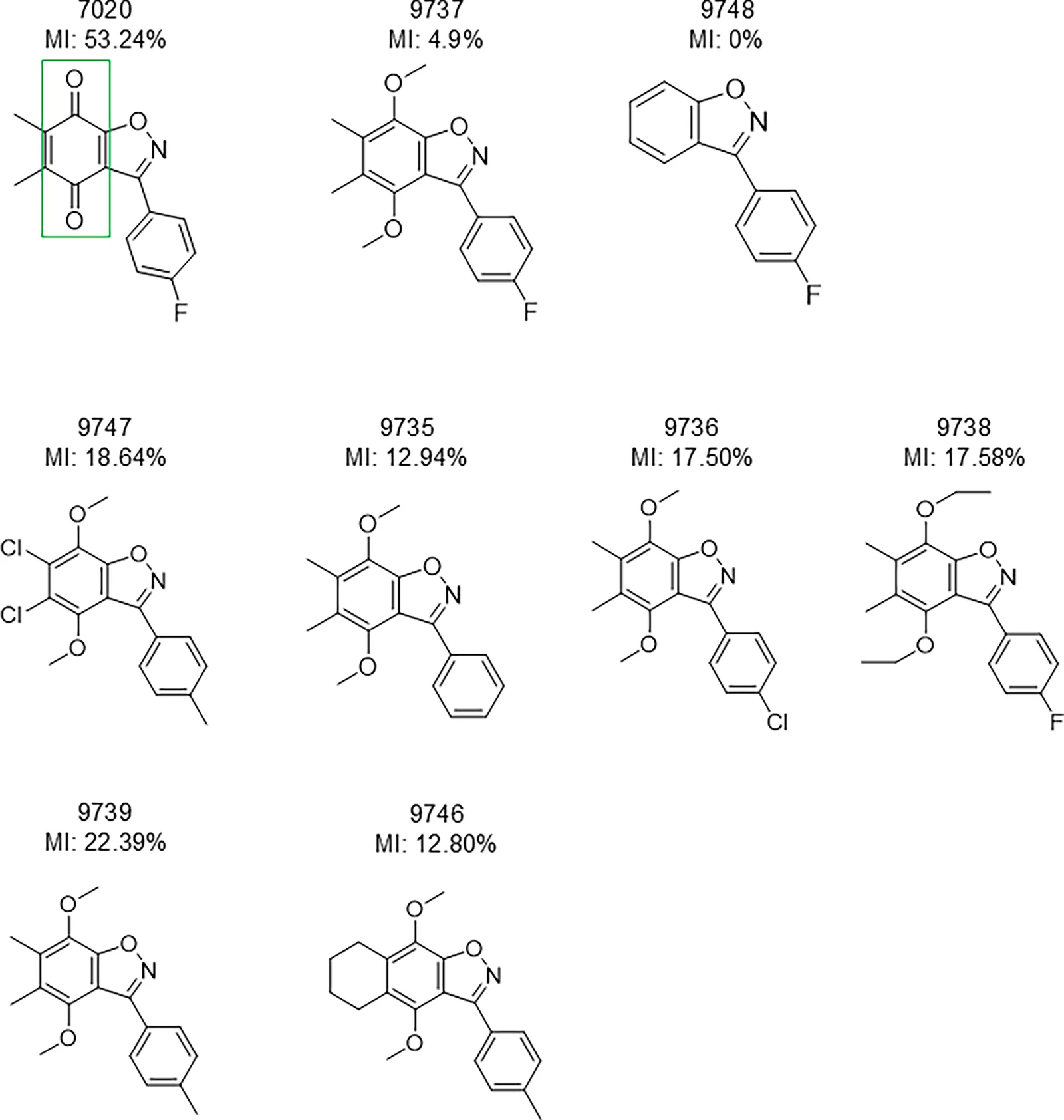
Analogs of 7020 lacking the benzoquinone structure are poor inhibitors of mistranslation. The structure of analogs of 7020 synthesized without the benzoquinone structure (boxed in green on 7020) and their percent inhibition of mistranslation (MI) are shown alongside 7020.

**Fig 6:**
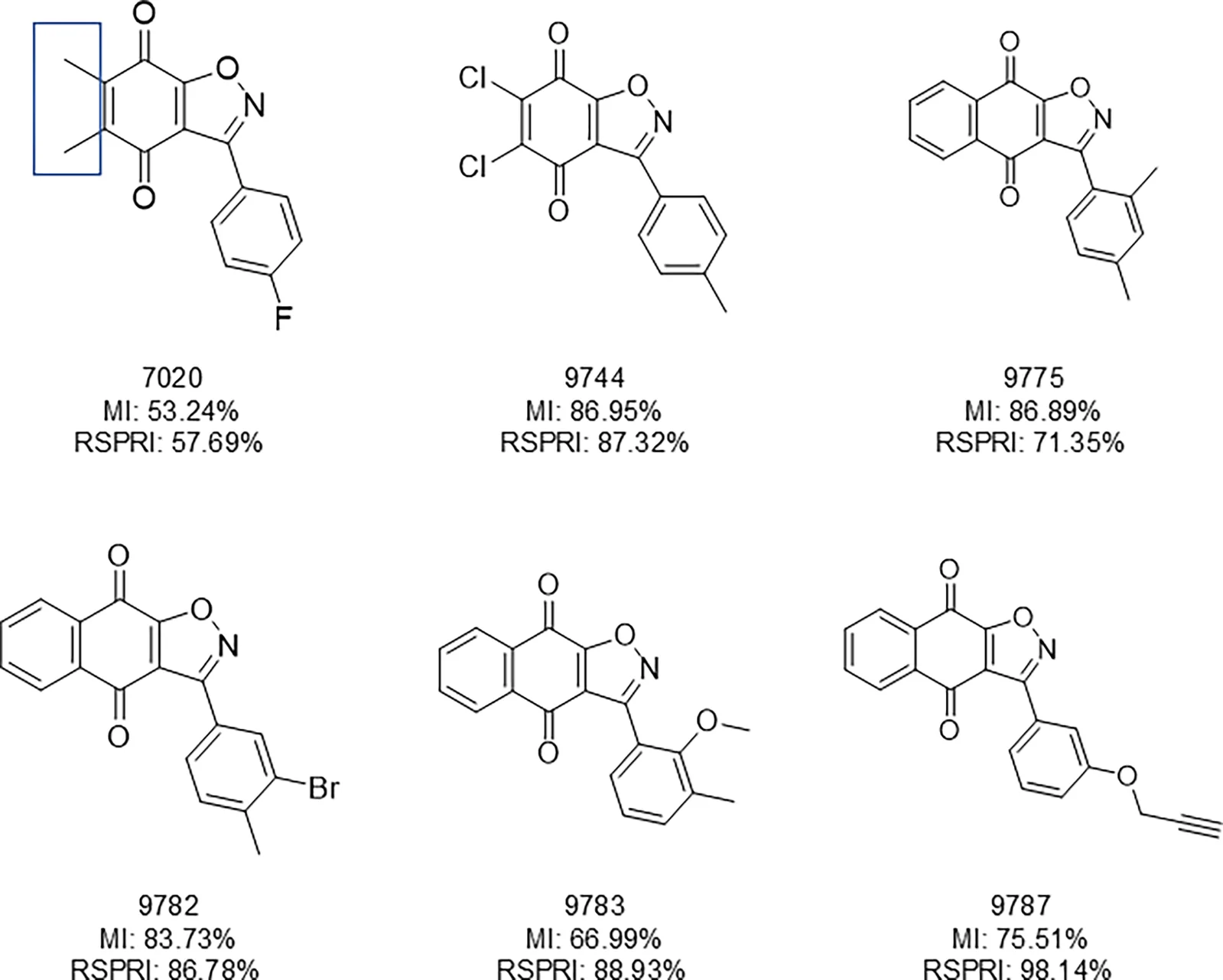
Replacement of 7020 methyl groups improves the bioactivity of analog compounds. The structures and percent inhibition of mistranslation and RSPR inhibition of 7020 analogs with improved bioactivity that have replacements of the methyl groups at positions 5 and 6 (boxed in blue on 7020) are shown alongside 7020. In 9744, the methyl groups have been replaced by chlorine. In 9775, 9782, 9783, and 9787, the methyl groups have been replaced by an aromatic ring.

Our preliminary investigation of structure-activity relationships (SARs) is summarized in Fig. 5 and 6. Our SAR analysis showed that the benzoquinone part of the compound is essential for its ability to reduce mistranslation; all analogs of 7020 synthesized without the benzoquinone structure are poor inhibitors of mistranslation (Fig. 5). We further observed that the methyl groups at positions 5 and 6 of the benzo[d]isoxazole-4,7-diones scaffold are not necessary for their bioactivities in terms of inhibition of mistranslation and RSPR (Fig. 6). Replacement of these methyl groups with other functional groups such as chlorine, as in 9744, or with an aromatic ring, as in 9775, 9782, 9783, and 9787, improved bioactivities of the compounds (Table 1; Fig. 6).

**Fig 7:**
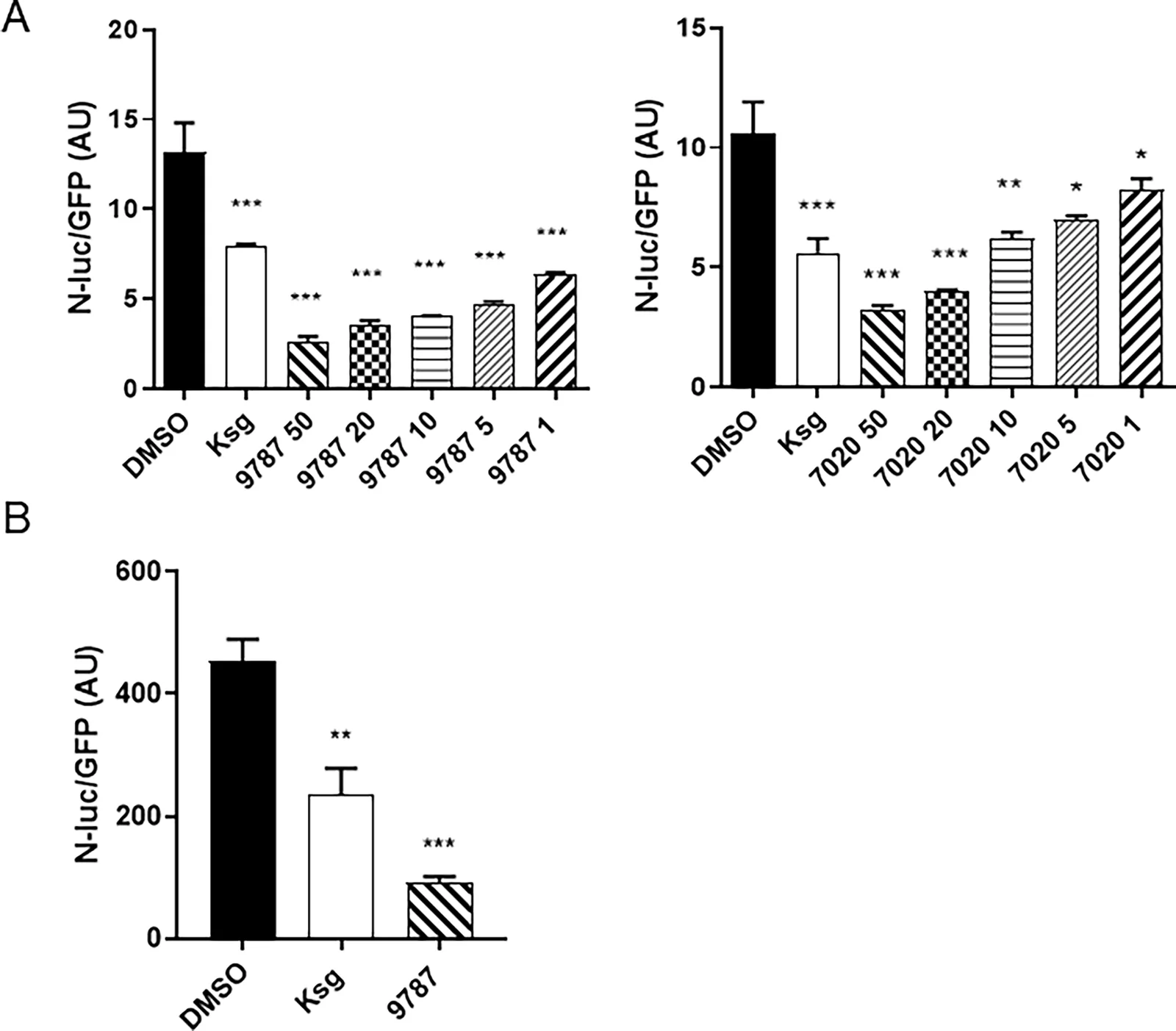
Compound 9787 decreases mistranslation more effectively than compound 7020 and decreases mistranslation in *M. bovis* BCG. (**A**) The N-luc/GFP reporter showed the activity of 9787 to inhibit mycobacterial mistranslation. Different doses of 9787 and the parent compound 7020 (in μg/mL) were used to check the improved potency of 9787. Dimethyl sulfoxide (DMSO) and kasugamycin (Ksg) were used as negative and positive controls, respectively. *P < 0.05, **P < 0.01, and ***P < 0.001 by Student’s *t*-test (**B**) Other than *M. smegmatis*, the N-luc/GFP mistranslation reporter could be expressed in *M. bovis* BCG strain. 9787 was able to decrease mistranslation also in BCG. **P < 0.01 and ***P < 0.001 by Student’s *t*-test.

We chose 9787 as the most promising candidate for attempting to determine the cellular target(s) of the hits. 9787 was more potent than the initial hit 7020 (Fig. 7A). Moreover, 9787 was able to decrease mistranslation in another closely related mycobacterial species, *M. bovis*-BCG (Fig. 7B), demonstrating activity across mycobacterial species.

### Chemical proteomics identifies ribosomal subunit protein S5 as the molecular target of 9787

**Fig 8:**
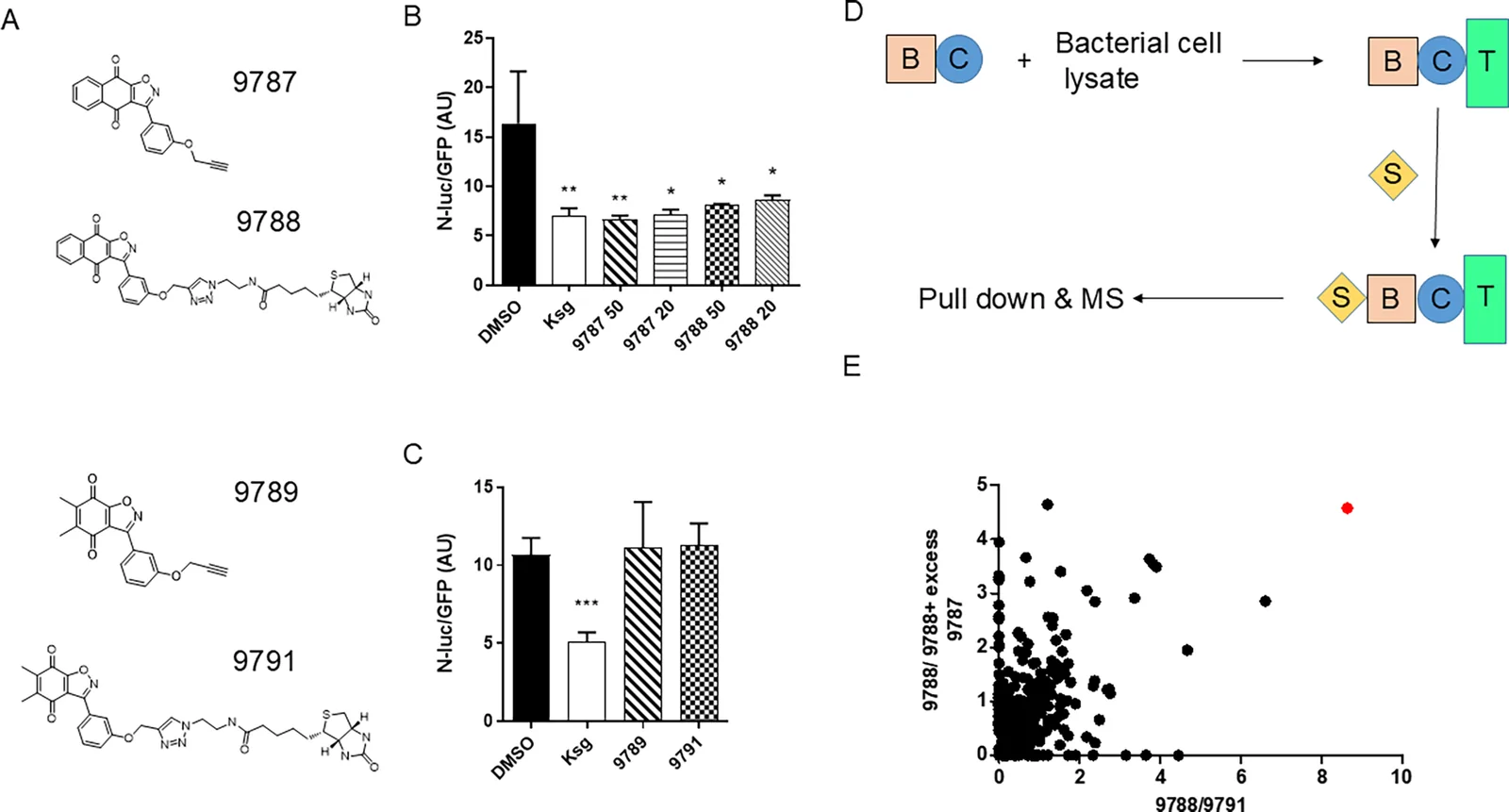
30S ribosomal protein S5 (RpS5) is the molecular target of compound 9787. (**A**) Structures of 9787, active biotinylated analog 9788, inactive analog 9789, and inactive biotinylated analog 9791. (**B**) The N-luc/GFP reporter was used to show the ability of 9787 and biotinylated 9788 (concentrations in μg/mL) to inhibit mycobacterial mistranslation. *P < 0.05, **P < 0.01, and ***P < 0.001 by Student’s *t*-test (**C**) 9789, unable to reduce mycobacterial mistranslation, was biotinylated to produce compound 9791. Neither of the compounds could inhibit mycobacterial mistranslation. (**D**) Schematic of the experiment for target identification of 9787. The compound of interest (**C**) is tagged with biotin (**B**) and incubated with mycobacterial cell lysate. After it binds to the target (T), the whole complex is pulled down using streptavidin (S) beads (see Materials and Methods for details). The pulled-down proteins are then analyzed by mass spectrometry. (**E**) The ratio of the quality scores of the proteins pulled down by the active biotinylated compound (9788) and the inactive biotinylated compound (9791) has been plotted against the ratio of the quality scores of proteins pulled down by the active alone (9788) and the mixture of 9788 and excess non-biotinylated active compound (9787). The 30S ribosomal protein S5 (Rps5) is represented by the red dot.

We wished to identify the cellular target by which 9787 mediated a decrease in specific mistranslation rates. Since the compounds lacked significant antimicrobial activity by themselves, it was not possible to raise mutants resistant to the compound and sequence for mutations in the genome to identify potential targets (22). We therefore decided to identify the target by a modified pull-down and mass-spectrometry technique (22–25). For this competitive chemical proteomic approach, 9787 was biotinylated to yield 9788, which retained activity (Fig. 8A and B). An inactive analog, 9789 from the initial medicinal chemistry approach was also biotinylated to make inactive 9791 (Fig. 8A and C). We incubated 9788 or 9791 with mycobacterial cell lysate. To increase the specificity of the identified target protein(s), we also included conditions in which fivefold molar excess of non-biotinylated 9787 or 9789 were added to the tubes in addition to the biotinylated compound (Fig. 8D and see Materials and Methods). Following incubation, the biotinylated compounds and putative cellular binding partners were pulled down using a streptavidin column, washed, and then the eluates were resolved on an SDS-PAGE gel and bands cut out and sent for analysis by mass spectrometry (Data S1). Our rationale for identifying the target for 9787 was as follows: when the active biotinylated compound (9788) was used, the quality score of the target protein should be significantly higher than that obtained with the inactive biotinylated compound (9791). Moreover, in the presence of competing non-biotinylated compounds, fewer target protein(s) are expected to bind to the streptavidin column. Hence, the quality score of the target protein obtained with the active 9788 alone should be significantly higher than that obtained with a mixture of 9788 and excess 9787. Furthermore, no such phenomenon should be observed in the case of the inactive compound (9789) and its biotinylated form (9791). We plotted the ratio of protein quality scores obtained with the active and inactive biotinylated compounds (9788/9791) against the ratio of protein quality scores obtained with 9788 alone, and 9788 and excess 9787 (9788/[9788 + excess 9787]). Out of 1,925 proteins, only the 30S ribosomal protein S5 (Rps5), derived from the *rpsE* gene could fulfill all the criteria and significantly stood out against the others (Fig. 8E). These data strongly suggest that RpS5 is the target of active 9787.

### Compound 9787 increases translational fidelity without affecting translation rate

**Fig 9:**
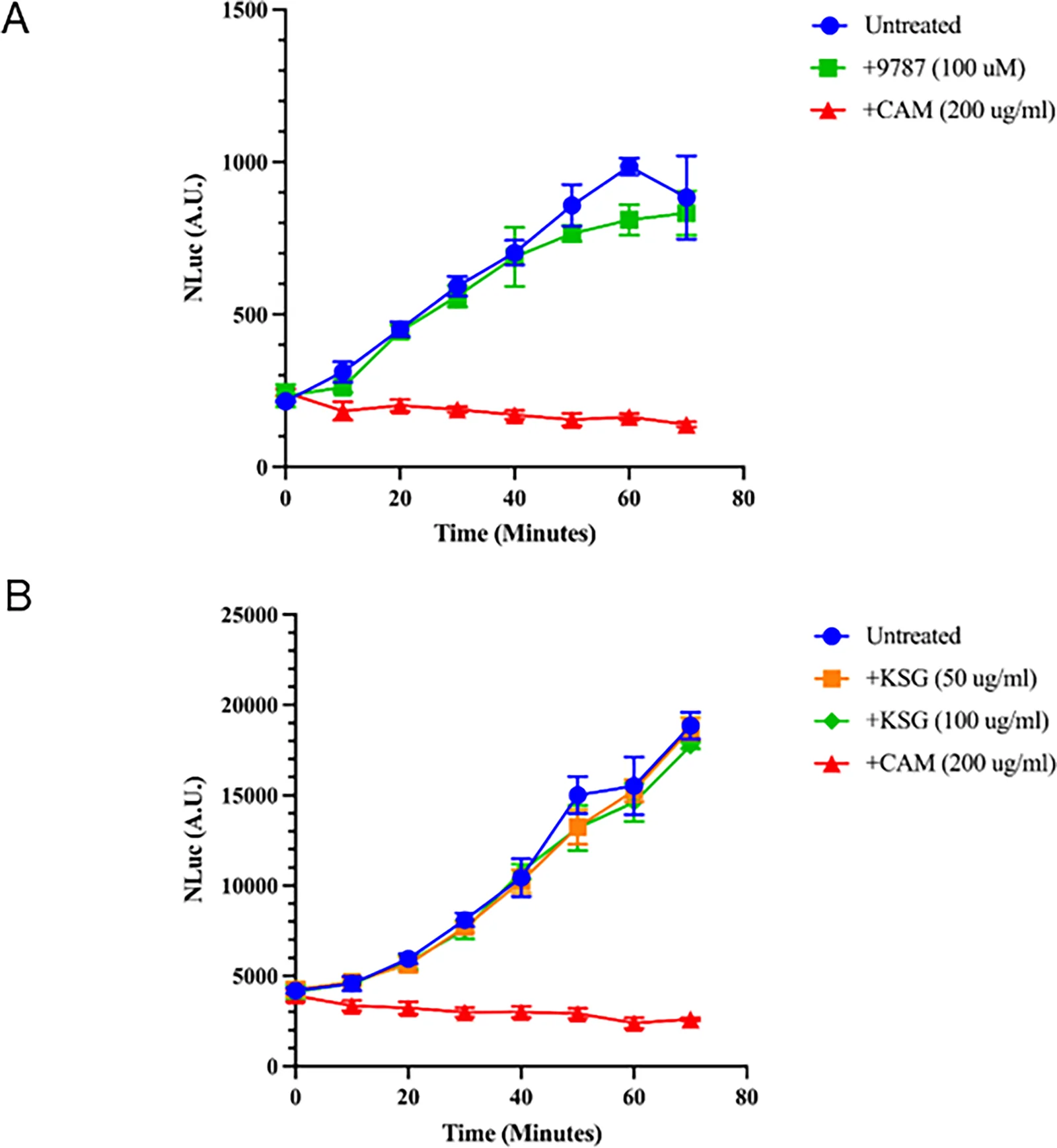
Neither 9787 nor kasugamycin alters translational efficiency in *M. smegmatis*. A tetracycline-inducible Nanoluc luciferase (Nluc) was induced with anhydrotetracycline at time = 0. Nluc activity was monitored over time as a proxy for translation rate in untreated *M. smegmatis,* and *M. smegmatis* treated with chloramphenicol (200 µg/mL) as a positive control for protein synthesis inhibition and 9787 (100 µg/mL) (**A**) or kasugamycin (50 µg/mL) (**B**). Experiments were performed in triplicate using three independent biological replicates.

A fundamental dogma in translation biology holds that increased translational fidelity requires decreased translation rate, that is, the “speed-accuracy trade-off” (26, 27). This model derives primarily from studies of codon-anticodon proofreading in *E. coli* and has been challenged in some studies (28, 29). To test if this applies to the discrimination of mischarged tRNAs by 9787, we measured protein synthesis rates in *M. smegmatis* cultures. We compared untreated cultures to those treated with 9787 or with chloramphenicol, a known translation inhibitor. As expected, chloramphenicol dramatically reduced the rate of protein synthesis (Fig. 9). In contrast, cultures treated with 9787 or kasugamycin at concentrations known to enhance fidelity showed translation rates that were not significantly different from untreated controls (Fig. 9). This key result demonstrates that 9787 enhances translational fidelity through a mechanism that is independent of the overall translation rate, challenging the universality of the speed-accuracy trade-off.

## DISCUSSION

In this study, we identify a novel class of synthetic molecules, benzo[d]isoxazole-4,7-diones, that increase mycobacterial translational fidelity. Our work makes three significant contributions. First, we establish a new chemical scaffold for modulating a specific mistranslation pathway that contributes to antibiotic tolerance. Second, using an unbiased chemical proteomics approach, we identify the 30S ribosomal protein S5 (RpS5) as the molecular target of our lead compound, 9787. Third, we demonstrate that this compound can enhance translational fidelity without a concomitant decrease in the rate of protein synthesis, providing a clear exception to the canonical “speed-accuracy trade-off” model of translation.

The “speed-accuracy trade-off” has been considered a fundamental constraint on ribosomal function (26, 27, 30). This model proposes that ribosomes sacrifice speed to achieve high fidelity through kinetic proofreading of codon-anticodon interactions. Our finding that 9787 enhances fidelity without a measurable impact on translation rate suggests this trade-off is not absolute, but comes with caveats with regard to over-interpretation. Using a luciferase reporter assay, we observed that compound 9787 enhanced fidelity without producing a measurable impact on the rate of active protein accumulation. We must, however, interpret this result with caution. The assay used is an indirect measure of translation, reflecting the combined rates of transcription, translation, and protein folding, and may lack the sensitivity to detect subtle but physiologically relevant changes in global translation rates. Nonetheless, this observation has several important implications. It suggests that the quality control mechanisms governing discrimination of mischarged tRNAs may be mechanistically distinct from those governing codon-anticodon matching. This is in keeping with prior studies that showed, in *E. coli* at least, the ribosome does not robustly discriminate against mischarged tRNAs when cognate codon-anticodon pairing has been established (31, 32). Our results reveal a potentially unexpected plasticity in ribosome function, where fidelity can be modulated without the obligate fitness cost of slowed protein synthesis. However, it may also be that cognate codon-anticodon discrimination is the major rate-limiting step in protein synthesis, and that other sources of error discrimination do not substantially impact speed. Further mechanistic, in particular kinetic studies, are needed to determine whether this is the case. Third, from a potential therapeutic perspective, it shows that translational fidelity can be pharmacologically modulated without imposing the fitness cost of slowed protein synthesis. Notably, small molecules have now been identified that can both positively (kasugamycin [19; this work]) and negatively (streptomycin [33]) influence translational fidelity in bacteria.

Our competitive chemical proteomic approach strongly suggests that the 30S ribosomal protein S5 (RpS5) is the molecular target of 9787. The use of active and inactive probes, along with competition with the parent compound, provides a high degree of confidence in this result. RpS5 is a key component of the 30S subunit’s decoding center and has a well-established role in maintaining translational fidelity, making it a highly plausible target for a fidelity-enhancing compound. We acknowledge, however, that this evidence is based on a single methodology. Future studies employing orthogonal validation techniques, such as the cellular thermal shift assay, would be necessary to definitively confirm a direct physical interaction and formally validate RpS5 as the target

RpS5 plays crucial roles in mRNA binding to the ribosome and proper decoding (34). Our results suggest that small molecule binding to RpS5 can enhance discrimination of mischarged aminoacyl-tRNAs. How might the small ribosomal subunit, primarily concerned with decoding, contribute to aminoacyl identity discrimination? The mechanism remains unknown, but we speculate that binding of 9787 to RpS5 induces a conformational change that allosterically tightens quality control at the A-site, enhancing rejection of near-cognate or misacylated tRNAs without affecting the rate-limiting steps of translation. Several lines of evidence support a role for the small subunit in this process. First, kasugamycin, our previously identified mistranslation inhibitor (19), also targets the 30S subunit, binding near residues A794 and G926 of 16S rRNA (35, 36). The universally conserved nature of these residues suggests kasugamycin likely binds similarly in mycobacteria. Thus, the 30S ribosomal subunit appears to be a common target for mistranslation inhibitors. Second, while the ribosome’s proofreading of codon-anticodon interactions is well-established (37), its ability to detect mischarged tRNAs is less understood. Our results suggest the 30S subunit can discriminate aminoacyl identity, possibly through conformational sensing mechanisms that are independent of the kinetic proofreading governing codon-anticodon fidelity. Third, the lack of impact of 9787 on translation rate suggests that it acts independently of peptide bond formation or aa-tRNA accommodation, a rate-limiting step in protein synthesis. Future studies, including structural or kinetic approaches, will be crucial in understanding this mechanism.

Although maintaining translational fidelity is believed to be essential for all living systems, bacteria have been shown to tolerate from 10% to 20% rates of mistranslation of a specific codon without major deleterious effects (38, 39). However, distinct types of mistranslation and differing cellular contexts may alter the “tolerability” of mistranslation rates (29, 40–42). Moreover, mistranslation has been proposed to be a mechanism by which bacteria such as *Mycobacterium* adapt to environmental stresses such as low pH and presence of rifampicin. Under such conditions, mistranslation results in increased phenotypic heterogeneity and improves bacterial survival (29, 43).

Rifampicin is a crucial antibiotic in anti-TB chemotherapy. Its introduction in TB therapy has drastically reduced the treatment time. However, due to persistence of the bacteria, the current treatment time for drug-susceptible TB is still 4–6 months, and thus has room to improve. Empirical attempts have been made to improve the efficacy of rifampicin by changing the therapeutic concentrations, as well as by using new combinations with other drugs (44–46). We, for the first time, had earlier proposed a mechanism of enhancing the efficacy of rifampicin in TB treatment by targeting mycobacterial mistranslation (19). In this work, we have further expanded our search for compounds that can decrease mycobacterial mistranslation. The “hits” have the potential to be used as candidates for combination therapy with rifampicin in the future. Importantly, this gives us confidence in using our platform to develop future drug candidates. Moreover, the 30S ribosomal subunit can be used as an attractive drug target for reducing the burden of antibiotic-tolerant bacteria.

Phenotypic screening has been extensively used in developing novel drug candidates against *Mycobacterium tuberculosis* (47–50). Phenotypic screening is in general considered a better approach than biochemical screening, which often yields ‘hits’ that cannot actually penetrate into the bacterial cell. This is particularly true for mycobacteria that have a complex capsule and cell wall. On the other hand, hits obtained from whole-cell phenotypic screening exhibit their effect after penetrating into the bacterial cell. Hence, they are usually better candidates for drug development than those obtained from biochemical screening.

In this study, we have successfully expanded one of the luciferase-based mycobacterial mistranslation assays (19) developed in our laboratory into a medium-scale screening platform. Our study was initiated with a whole-cell phenotypic screen to identify novel modulators of mycobacterial mistranslation. It is important to acknowledge the limitations of this initial screen. The assay was designed to detect a reduction in an inherently low and noisy baseline signal (~1% mistranslation), which resulted in variable performance, with nearly a third of plates yielding a *Z*′-factor below our quality threshold. While this presents a challenge for confidently identifying all potential hits, the screen was nonetheless successful in identifying the benzo[d]isoxazole-4,7-dione scaffold, which was subsequently validated through re-synthesis and secondary assays. Therefore, while the screen itself was not optimally robust for a large-scale campaign, it served as an effective discovery tool that provided a valuable and chemically tractable starting point for our investigation.

Several important questions remain. (i) Do these compounds affect other types of translational errors beyond mischarged tRNAs? (ii) What is the structural basis for RpS5-mediated fidelity enhancement? (iii) Can these compounds or optimized derivatives show efficacy in animal models of tuberculosis?

In conclusion, we have identified synthetic small molecules that increase mycobacterial translational fidelity through a rate-independent mechanism by targeting ribosomal protein S5. This work challenges prevailing models of ribosomal quality control, reveals new therapeutic strategies for antibiotic-tolerant bacteria, and demonstrates that the 30S ribosomal subunit is an attractive drug target for reducing the burden of persistent mycobacterial infections.

## MATERIALS AND METHODS

### Bacterial strains, culture, antibiotics, and test compounds

Wild-type *M. smegmatis* mc^2^-155 and its mistranslation reporter derivative strain were grown in Middlebrook 7H9 broth supplemented with 0.2% glycerol, 0.05% Tween-80, 10% albumin-dextrose-salt (ADS), and on Luria-Bertani agar (LB agar) for plate assays. Rifampicin and kasugamycin (Ksg) were purchased from Sigma. Rifampicin was dissolved in dimethyl sulfoxide (DMSO); Ksg was dissolved in water, and filter sterilized. The test compounds for screening were all dissolved in DMSO.

### Screening of compounds that decrease mycobacterial mistranslation

Screening of the chemical library to identify compounds that decrease mycobacterial mistranslation was done using the previously developed N-luc/GFP mistranslation assay (11). Briefly, the mistranslation reporter *M. smegmatis* strain or *M. bovis* BCG strain was grown in 7H9 broth containing hygromycin (50 µg/mL) and kanamycin (20 µg/mL) at 37°C to late log phase (OD_600_ = 2.0). N-luc expression was induced by adding anhydrotetracycline (ATc, 50 ng/mL), and the bacterial culture was immediately aliquoted into a 96-well plate (100 µL, i.e., approximately 6 × 10^7^ cells per well). Fifty micromolars of each of the test compound or 50 µg/mL of Ksg as positive control was added, and the cultures incubated for 16 h at 37˚C with shaking. Then, the cultures were transferred to a 96-well black plate, and GFP fluorescence was measured by Fluoroskan Ascent FL Fluorometer and Luminometer (Thermo Scientific). The plate was then centrifuged (4,000 rpm for 10 min), and supernatants transferred to a 96-well white luminescence plate. The nano-luciferase assay was performed using the Nano-Glo luciferase assay kit (Promega), and luminescence was measured by the same machine.

### Rifampicin-specific phenotypic resistance (RSPR) assay

RSPR assay was performed as previously described (19). Briefly, *M. smegmatis* mc^2^-155 was grown to stationary phase, after which the cultures were serially diluted and spread on LB agar plates containing rifampicin (50 µg/mL) only, or rifampicin and 50 µM of each test compound. LB agar containing rifampicin and Ksg (100 µg/mL) was used as positive control. The plates were incubated at 37°C for 5 days, after which the number of colony-forming units (cfu) was enumerated. The number of bacterial cells in the inoculum was calculated by counting colonies after spreading serial dilutions of the culture on LB-agar plates without any antibiotic.

### Target identification of 9788

To identify the intracellular target(s) of compounds that decrease mycobacterial mistranslation, 9788, a biotinylated version of compound 9787 that decreased mycobacterial mistranslation, and 9791, a biotinylated version of a compound 9789 that cannot decrease mycobacterial mistranslation, were used. A protocol modified from an earlier study to identify mycobacterial drug targets was used (51). To prepare mycobacterial cell lysate, *M. smegmatis* mc^2^-155 was grown in 7H9 broth up to OD_600_ of 1.0, and then centrifuged at 4,000 rpm for 10 min. The pellet was resuspended in phosphate-buffered saline (PBS) containing a tablet of protease inhibitor (Roche), and the cells were lysed by beads beating twice for 45 s each. Then, the mixture was centrifuged at 15,000 rpm for 15 min, the supernatant was collected and added to EZ view Red streptavidin affinity gel (Sigma-Aldrich), pre-equilibrated with the lysis buffer (PBS plus protease inhibitor cocktail). The mixture was incubated at 4°C for an hour in an orbital shaker, and then centrifuged at 15,000 rpm for an hour to collect the supernatant. The supernatant was then distributed to five tubes, and the protein concentration of each tube was adjusted to 1 mg/mL. The following compounds were then added one to each tube: (i) 1% DMSO, (ii) 100 μM 9788, (iii) 100 μM 9788 + 500 µM 9787, (iv) 100 μM 9791, and (v) 100 μM 9791 + 500 µM 9789. After 1-h incubation at room temperature, the mixture from each tube was added to pre-equilibrated EZ view Red streptavidin affinity gel, and incubated at 4°C for an hour in an orbital shaker. Then, the mixtures were centrifuged at 15,000 rpm for 20 min at 4°C. The pellets were then resuspended in Laemmli buffer, run in SDS-PAGE, and sent for mass spectrometry at the Tsinghua University Mass Spectrometry Facility.

### Relative translation rate assay

To assess the relative translation rate, we adapted a previously described protocol (52) with minor modifications. *M. smegmatis* strain harboring the reporter construct was first grown in 7H9 medium to a stationary phase (OD_600_ > 3). Cultures were then diluted to an OD_600_ of 0.2 in fresh 7H9 medium, and anhydrotetracycline (ATc) was added to a final concentration of 50 ng/mL to induce reporter expression. After 5 min of induction, cells were pelleted, washed twice with fresh 7H9 medium (without ATc), and resuspended in the same medium supplemented with or without 200 μg/mL chloramphenicol (a translation inhibitor) and 100 μg/mL compound 9787. Higher concentrations of 9787 could not be tested since the stock solution was 10 mg/mL dissolved in DMSO and final DMSO concentration needed to be ≤1%. Aliquots were collected every 10 min and immediately snap-frozen in liquid nitrogen. Once all time points were collected, luminescence was measured using a luminometer. All experiments were performed in triplicate using three independent biological replicates.

## SUPPLEMENTAL MATERIAL

Data S1 mbio.02056-26-s0001.xlsx Chemical proteomics.

Supplemental information mbio.02056-26-s0002.pdf Supplemental text.

ASM does not own the copyrights to Supplemental Material that may be linked to, or accessed through, an article. The authors have granted ASM a non-exclusive, world-wide license to publish the Supplemental Material files. Please contact the corresponding author directly for reuse.
