# Supplementary material for: A small molecule increases mycobacterial translation fidelity without a fitness cost by targeting ribosomal protein S5
Source: mBio. 2026 Oct 2;17(11):e02056-26. doi: 10.1128/mbio.02056-26 (PMC13753742; doi:10.1128/mbio.02056-26)
Supplement: Supplementary file 1 [file mbio.02056-26-s0002.pdf]

Supporting Information: The experimental procedures of chemistry and spectroscopic data of all compounds.

Title: A small molecule increases mycobacterial translation fidelity without a fitness cost by targeting ribosomal protein S5

Authors: Jie Wu[1], Swarnava Chaudhuri[2], Sarah Caldwell Feid[3], Miaomiao Pan[2], Qingwen Kawaji[2], Gang Liu[1*] and Babak Javid[2, 3*]

JW, SC and SCF contributed equally to this work

GL and BJ contributed equally to this work

[*]To whom correspondence should be addressed: babak.javid@ucsf.edu (BJ) or gangliu27@tsinghua.edu.cn (GL)

Running title: Benzo[d]isoxazole-4,7-dione analogues decrease mycobacterial mistranslation

MATERIALS

Unless otherwise noted, commercially available materials were used without further purification. All NMR experiments were carried out on a Bruker Advance 400 MHz spectrometer using DMSO-d_6, methanol-d_4, Acetone-d_6 and $CDCl_3$ as the solvent. Chemical shifts are given in ppm (δ) relative to the residual solvent and coupling constants (J) in Hz. Multiplicity is tabulated as s for singlet, d for doublet, t for triplet, q for quadruplet, and m for multiplicity. Melting points were determined without correction with a Yanaco micromelting point apparatus. UPLC-MS analysis was performed on a Thermo Finnigan LCQ Advantage mass spectrometer equipped with an Agilent pump, an Agilent detector, an Agilent liquid handler, and a fluent splitter. The column is a Kromasil C18 column (4.6 µm, 4.6 mm × 50 mm). The eluent was a mixture of acetonitrile and water containing 0.05% HCOOH with a linear gradient from 5:95 (v/v) to 95:5 (v/v) of acetonitrile-H_2O within 5 minutes at a 0.3 mL/min flow rate for analysis. The UV detection was carried out at a UV wavelength of 254 nm. The 5% of the eluent was split into the MS system. Mass spectra were recorded in either positive or negative ion mode using electrospray ionization (ESI). High resolution LC-MS (HRMS) was carried out by Agilent LC/ MSD TOF using a column of Agilent ZORBAX SB-C18 (rapid resolution, 3.5 µm, 2.1 mm × 30 mm) at a flow of 0.40 mL/min. The solvent was methanol/water = 75/25 (v/v) containing 5 mmol/L ammonium formate. The ion source was electrospray ionization (ESI). The column chromatography was performed with silica gel 60 (200-300 mesh) from Qingdao Haiyang Chemical Factory. The tested compounds were purified ≥ 95%, as characterized by HPLC under UV 254 nm wavelength, NMR and HRMS.

EXPERIMENTAL PROCEDURES OF CHEMISTRY AND SPECTROSCOPIC DATA

General Procedure one: To a solution of aldehyde (10.0 mmol) in methanol (50 mL) was added potassium carbonate (11.0 mmol) and hydroxylamino hydrochloride (11.0 mmol) at room temperature. The mixture was stirred at room temperature for 3 h until the reaction was complete. The solution was evaporated in vacuo and the residue was stirred in ethyl acetate (50.0 mL) for 10 minutes. The solution was filtered and the filtrate was evaporated to get the intermediate aldehyde oxime.

To a solution of phenol (2.0 mmol) in acetonitrile (10 mL) and water (5 mL) was added $PhI(OAc)_2$ (4.0 mmol) at room temperature. The mixture was stirred at room temperature until the starting material disappeared. Then aldehyde oxime (3.0 mmol) was added. After the flask was completely cooled in an ice bath, 4.0 mmol of $PhI(OAc)_2$ solved in acetonitrile (8 mL) and water (2mL) was added dropwise within 0.5 h and then the mixture warmed to room temperature. After the intermediate disappeared, the mixture was poured into brine (60 mL) and extracted with ethyl acetate (50 mL × 3). The combined organic layer was washed with brine (50 mL × 2) and dried over Na_2SO_4. After evaporation of the solvent in vacuo, the compound was obtained and characterized after purification by silica gel column chromatography (ethyl acetate/petroleum ether).

5,6-dimethyl-3-phenylbenzo[d]isoxazole-4,7-dione (7006). Yellow powder in 80.1% yield. ^{1}H-NMR (400 MHz, $CDCl_3$) δ 8.11 (d, J = 7.5 Hz, 2H), 7.49-7.56 (m, 3H), 2.17 (s, 6H). ^{13}C-NMR (100 MHz, $CDCl_3$) δ180.7, 175.3, 164.9, 160.2, 144.1, 140.1, 131.2, 129.2, 128.6, 126.2, 117.5, 13.10, 12.04. ESI-MS: calcd for $C_{15}H_{11}NO_3$ $[M+H]^+$: 254.12; found, 254.07.

3-(4-chlorophenyl)-5,6-dimethylbenzo[d]isoxazole-4,7-dione (7013). Yellow powder in 85.0% yield. ^{1}H-NMR (400 MHz, $CDCl_3$) δ 8.12 (d, J = 8.4 Hz, 2H), 7.49 (d, J = 8.4 Hz, 2H), 2.17 (s,

6H). ^{13}C-NMR (100 MHz, $CDCl_3$) δ 180.7, 175.1, 165.0, 159.2, 144.1, 140.3, 137.5, 130.5, 129.0, 124.7, 117.4, 13.1, 12.1. ESI-MS: calcd for $C_{15}H_{10}ClNO_3$ $[M+H]^+$: 288.19; found, 288.03.

3-(4-fluorophenyl)-5,6-dimethylbenzo[d]isoxazole-4,7-dione (7020). Yellow powder in 69.8% yield. ^{1}H-NMR (400 MHz, $CDCl_3$) δ 8.18 (dd, J = 8.6 Hz, 5.5 Hz, 2H), 7.20 (t, J = 8.6 Hz, 2H), 2.17 (s, 6H). ^{13}C-NMR (100 MHz, $CDCl_3$) δ 180.8, 175.2, 165.8, 165.0, 163.3, 159.2, 144.1, 140.3, 131.5, 131.4, 122.4, 122.4, 117.3, 116.0, 115.8, 13.1, 12.1. ESI-MS: calcd for $C_{15}H_{10}FNO_3$ $[M+H]^+$: 272.54; found, 272.06.

5,6-dimethyl-3-(naphthalen-1-yl)benzo[d]isoxazole-4,7-dione (9719). Yellow powder in 61.4% yield. ^{1}H-NMR (400 MHz, $CDCl_3$) δ 8.04 (d, J = 8.2 Hz, 1H), 7.94 (d, J = 8.1 Hz, 1H), 7.86 (d, J = 8.3 Hz, 1H), 7.73 (d, J = 7.1 Hz, 1H), 7.61 – 7.49 (m, 3H), 2.17 (s, 3H), 2.07 (s, 3H). ^{13}C-NMR (100 MHz, $CDCl_3$) δ 180.3, 175.4, 164.3, 159.4, 143.8, 140.5, 133.6, 131.3, 131.1, 129.1, 128.6, 127.2, 126.5, 125.1, 124.9, 123.3, 118.9, 12.9, 12.2. ESI-MS: calcd for $C_{19}H_{13}NO_3$ $[M+H]^+$: 304.51; found, 304.09.

3-(4-fluorophenyl)-5,6-dimethyl-1H-indazole-4,7-dione (9741). Yellow powder in 64.1% yield. ^{1}H-NMR (400 MHz, DMSO-d_6) δ 14.55 (s, 1H), 8.15 (s, 2H), 7.37 (s, 2H), 2.03 (s, 6H). ^{13}C-NMR (100 MHz, DMSO-d_6) δ 181.3, 179.6, 164.6, 162.1, 144.8, 140.9, 130.7, 130.6, 125.2, 115.4, 115.3, 115.1, 13.1, 12.2. ESI-MS: calcd for $C_{15}H_{11}FN_2O_2$ $[M+H]^+$: 271.37; found, 271.08.

5,6-dichloro-3-(p-tolyl)benzo[d]isoxazole-4,7-dione (9744). Yellow powder in 53.1% yield. ^{1}H-NMR (400 MHz, $CDCl_3$) δ 7.95 (d, J = 8.2 Hz, 1H), 7.74 (d, J = 8.2 Hz, 1H), 7.31 (dd, J = 8.0 Hz, 2.9 Hz, 2H), 2.44 (d, J = 2.7 Hz, 3H). ^{13}C-NMR (100 MHz, $CDCl_3$) δ 170.3, 169.5, 159.9, 159.3, 143.5, 142.0, 129.8, 129.4, 129.2, 127.3, 117.4, 21.6. ESI-MS: calcd for $C_{14}H_7Cl_2NO_3$ $[M+H]^+$: 308.36; found, 307.98.

3-(o-tolyl)naphtho[2,3-d]isoxazole-4,9-dione (9759). Yellow powder in 83.4% yield. ^{1}H-NMR (400 MHz, $CDCl_3$) δ 8.36-8.28 (m, 1H), 8.24-8.19 (m, 1H), 7.90-7.83 (m, 2H), 7.48 (dd, J = 12.2 Hz, 4.5 Hz, 2H), 7.44-7.33 (m, 2H), 2.37 (s, 3H). ^{13}C-NMR (100 MHz, $CDCl_3$) δ 178.5, 173.4, 165.4, 160.8, 137.6, 135.2, 134.3, 133.5, 132.1, 130.66, 130.65, 130.2, 127.49, 127.43, 125.7, 125.5, 120.6, 20.2. ESI-MS: calcd for $C_{18}H_{11}NO_3$ $[M+H]^+$: 290.26 found, 290.07.

3-(naphthalen-1-yl)naphtho[2,3-d]isoxazole-4,9-dione (9761). Yellow powder in 93.1% yield. ^{1}H-NMR (400 MHz, $CDCl_3$) δ 8.36-8.30 (m, 1H), 8.15 (dt, J = 6.6 Hz, 3.1 Hz, 1H), 8.08 (d, J = 8.3 Hz, 1H), 7.97 (d, J = 7.7 Hz, 1H), 7.89 (d, J = 8.4 Hz, 1H), 7.87-7.76 (m, 3H), 7.63 (dd, J = 8.2 Hz, 7.2 Hz, 1H), 7.51-7.57 (m, 2H). ^{13}C-NMR (100 MHz, $CDCl_3$) δ 178.2, 173.4, 165.6, 160.2, 135.3, 134.3, 133.6, 133.5, 132.1, 131.4, 131.2, 129.1, 128.6, 127.5, 127.4, 127.2, 126.5, 125.0, 124.9, 123.2, 121.0. ESI-MS: calcd for $C_{21}H_{11}NO_3$ $[M+H]^+$: 326.58 found, 326.07.

3-cyclohexylnaphtho[2,3-d]isoxazole-4,9-dione (9767). Yellow powder in 89.3% yield. ^{1}H-NMR (400 MHz, $CDCl_3$) δ 8.29-8.19 (m, 2H), 7.87-7.79 (m, 2H), 3.26-3.30 (m, 1H), 2.09 (d, J = 11.4 Hz, 2H), 1.93-1.86 (m, 2H), 1.81-1.67 (m, 3H), 1.40-1.46 (m, 3H). ^{13}C-NMR (100 MHz, $CDCl_3$) δ 179.4, 173.4, 166.3, 165.3, 135.1, 134.2, 133.5, 132.1, 127.4, 127.3, 120.1, 36.1, 30.5, 26.0, 25.7. ESI-MS: calcd for $C_{17}H_{15}NO_3$ $[M+H]^+$: 282.34 found, 282.11.

3-(2,4-dimethylphenyl)naphtho[2,3-d]isoxazole-4,9-dione (9775). Yellow powder in 81.2% yield. ^{1}H-NMR (400 MHz, $CDCl_3$) δ 8.29 (dd, J = 6.0 Hz, 2.9 Hz, 1H), 8.22-8.15 (m, 1H), 7.87-7.81 (m, 2H), 7.38 (d, J = 7.8 Hz, 1H), 7.21-7.11 (m, 2H), 2.41 (s, 3H), 2.32 (s, 3H). ^{13}C-NMR (100 MHz, $CDCl_3$) δ 178.5, 173.4, 165.4, 160.8, 140.7, 137.4, 135.2, 134.2, 133.6, 132.1, 131.5, 130.2, 127.4, 127.3, 126.5, 122.5, 120.6, 21.4, 20.1. ESI-MS: calcd for $C_{19}H_{13}NO_3$ $[M+H]^+$: 304.54 found, 304.09.

3-(3-bromo-4-methylphenyl)naphtho[2,3-d]isoxazole-4,9-dione (9782). Yellow powder in 73.1% yield. ^{1}H-NMR (400 MHz, $CDCl_3$) δ 8.42 (s, 1H), 8.29 (dd, J = 7.4 Hz, 1.4 Hz, 2H), 8.06 (dd, J = 7.9 Hz, 1.4 Hz, 1H), 7.90-7.82 (m, 2H), 7.41 (d, J = 7.9 Hz, 1H), 2.50 (s, 3H). ^{13}C-NMR (100 MHz, $CDCl_3$) δ 178.5, 173.2, 166.3, 159.6, 141.5, 135.3, 134.3, 133.7, 132.9, 131.7, 130.9, 128.1, 127.8, 127.3, 125.4, 125.1, 119.5, 23.1. ESI-MS: calcd for $C_{18}H_{10}BrNO_3$ $[M+H]^+$: 368.02 found, 367.98.

3-(2-methoxy-3-methylphenyl)naphtho[2,3-d]isoxazole-4,9-dione (9783). Yellow powder in 43.6% yield. ^{1}H-NMR (400 MHz, $CDCl_3$) δ 8.30-8.10 (m, 2H), 7.90-7.72 (m, 2H), 7.46-7.32 (m, 2H), 7.32-7.16 (m, 1H), 3.99 (s, 3H), 2.66 (s, 3H). ^{13}C-NMR (100 MHz, $CDCl_3$) δ 183.0, 180.7, 162.4, 159.3, 151.4, 134.6, 133.7, 131.7, 131.2, 130.4, 126.7, 124.3, 122.2, 120.9, 119.3, 117.1, 61.8, 15.2. ESI-MS: calcd for $C_{19}H_{13}NO_4$ $[M+H]^+$: 320.18 found, 320.08.

3-(3-(prop-2-yn-1-yloxy)phenyl)naphtho[2,3-d]isoxazole-4,9-dione (9787). Yellow powder in 80.1% yield. ^{1}H-NMR (400 MHz, DMSO-d_6) δ 8.20 (d, J = 7.0 Hz, 2H), 7.99 (dt, J = 6.9 Hz, 5.7 Hz, 2H), 7.73 (d, J = 7.1 Hz, 2H), 7.57 (t, J = 8.0 Hz, 1H), 7.31 – 7.25 (m, 1H), 4.93 (d, J = 2.2 Hz, 2H), 3.64 (t, J = 2.1 Hz, 1H).^{13}C-NMR (100 MHz, $CDCl_3$) δ 178.5, 173.3, 166.3, 160.7, 157.6, 135.3, 134.3, 133.8, 131.7, 129.8, 127.8, 127.4, 127.2, 122.6, 119.6, 118.4, 115.5, 78.2, 75.8, 56.1. ESI-MS: calcd for $C_{20}H_{11}NO_4$ $[M+H]^+$: 330.24 found, 330.07.

5,6-dimethyl-3-(3-(prop-2-yn-1-yloxy)phenyl)benzo[d]isoxazole-4,7-dione (9789). Yellow powder in 81.1% yield. ^{1}H-NMR (400 MHz, $CDCl_3$) δ 7.62-7.36 (m, 2H), 7.29 (t, J = 3.0 Hz, 1H), 7.00-7.12 (m, 1H), 4.97 (d, J = 2.1 Hz, 2H), 2.61 (s, 1H), 2.12 (s, 3H), 2.17 (s, 3H). ^{13}C-NMR (100 MHz, $CDCl_3$) δ 178.5, 173.3, 166.3, 160.7, 157.6, 135.3, 134.3, 133.8, 131.7, 129.8, 127.8, 127.4, 127.2, 122.6, 119.6, 118.4, 115.5, 78.2, 75.8, 56.1. ^{13}C-NMR (100 MHz, $CDCl_3$) δ 183.7,

175.9, 161.5, 160.4, 156.0, 144.9, 143.6, 131.0, 128.7, 122.8, 116.5, 113.8, 112.6, 79.6, 76.8, 58.0, 12.7, 12.2. ESI-MS: calcd for $C_{18}H_{13}NO_4$ $[M+H]^+$: 308.53 found, 308.08.

General Procedure two: To a solution of benzo[d]isoxazole-4,7-dione (1.2 mmol) in dichloromethane (15.0 mL) was added $Na_2O_4S_2$ (7.1 mmol) in water (15.0 mL) at room temperature. The mixture was stirred at room temperature until the starting material disappeared. Then the mixture was poured into brine (60 mL) and extracted with dichloromethane (20 mL × 3). The combined organic layer was washed with brine (50 mL × 2) and dried over Na_2SO_4. After evaporation of the solvent in vacuo, the intermediate was dissolved in acetone (15 mL). Then K_2CO_3 (4.1 mmol) and CH_3I or CH_3CH_3I (4.7 mmol) were added. The mixture was stirred at room temperature for 3 h until the reaction was complete. The solution was filtered and the filtrate was evaporated to get the crude product. The compound was obtained and characterized after purification by silica gel column chromatography (ethyl acetate/petroleum ether).

4,7-dimethoxy-5,6-dimethyl-3-phenylbenzo[d]isoxazole (9735). White solid in 78.9% yield. ^{1}H-NMR (400 MHz, DMSO-*d$_6$*) δ 7.88 (dd, J = 7.1, 2.0 Hz, 2H), 7.63-7.56 (m, 3H), 4.03 (s, 3H), 3.24 (s, 3H), 2.29 (s, 3H), 2.22 (s, 3H). ^{13}C-NMR (100 MHz, DMSO-*d$_6$*) δ 157.5, 155.4, 146.4, 137.9, 132.2, 130.6, 129.4, 128.9, 128.5, 126.3, 113.8, 62.1, 60.9, 13.2, 12.5. ESI-MS: calcd for $C_{17}H_{17}NO_3$ $[M+H]^+$: 254.62; found, 284.12.

3-(4-chlorophenyl)-4,7-dimethoxy-5,6-dimethylbenzo[d]isoxazole (9736). White solid in 84.6% yield. ^{1}H-NMR (400 MHz, DMSO-*d$_6$*) δ 7.93 (d, J = 8.4 Hz, 2H), 7.66 (d, J = 8.4 Hz, 2H), 4.03 (s, 3H), 3.29 (s, 3H), 2.29 (s, 3H), 2.23 (s, 3H). ^{13}C-NMR (100 MHz, DMSO-*d$_6$*) δ 156.5, 155.4, 146.3, 137.9, 135.5, 132.4, 131.3, 129.1, 127.3, 126.5, 113.6, 62.2, 60.9, 13.2, 12.5. ESI-MS: calcd for $C_{17}H_{16}ClNO_3$ $[M+H]^+$: 318.52; found, 318.08.

3-(4-fluorophenyl)-4,7-dimethoxy-5,6-dimethylbenzo[d]isoxazole (9737). White solid in 84.9% yield. ^{1}H-NMR (400 MHz, DMSO-d_6) δ 7.95 (dd, J = 8.4 Hz, 5.6 Hz, 2H), 7.43 (t, J = 8.8 Hz, 2H), 4.03 (s, 3H), 3.27 (s, 3H), 2.29 (s, 3H), 2.22 (s, 3H). ^{13}C-NMR (100 MHz, DMSO-d_6) δ 164.9, 162.5, 156.6, 155.4, 146.3, 137.9, 132.3, 131.9, 131.8, 126.3, 124.9, 124.9, 116.2, 115.9, 113.7, 62.1, 60.5, 13.2, 12.5. ESI-MS: calcd for $C_{17}H_{16}FNO_3$ $[M+H]^+$: 302.18; found, 302.11.

4,7-diethoxy-3-(4-fluorophenyl)-5,6-dimethylbenzo[d]isoxazole (9738). White solid in 80.2% yield. ^{1}H-NMR (400 MHz, DMSO-d_6) δ 8.01-7.94 (m, 2H), 7.52-7.46 (m, 2H), 4.32 (q, J = 7.0 Hz, 2H), 3.46 (q, J = 7.0 Hz, 2H), 2.35 (s, 3H), 2.27 (s, 3H), 1.43 (t, J = 7.0 Hz, 3H), 1.00 (t, J = 7.0 Hz, 3H). ^{13}C-NMR (100 MHz, DMSO-d_6) δ 164.9, 162.5, 156.8, 155.6, 145.2, 136.7, 132.8, 132.1, 131.9, 126.6, 125.02, 124.99, 116.1, 115.9, 113.9, 70.9, 69.1, 15.9, 14.9, 13.6, 12.9. ESI-MS: calcd for $C_{19}H_{20}FNO_3$ $[M+H]^+$: 330.47; found, 330.14.

4,7-dimethoxy-5,6-dimethyl-3-(p-tolyl)benzo[d]isoxazole (9739). White solid in 71.2% yield. ^{1}H-NMR (400 MHz, DMSO-d_6) δ 7.79 (d, J = 8.1 Hz, 2H), 7.39 (d, J = 7.9 Hz, 2H), 4.02 (s, 3H), 3.26 (s, 3H), 2.42 (s, 3H), 2.28 (s, 3H), 2.22 (s, 3H). ^{13}C-NMR (100 MHz, DMSO-d_6) δ 157.4, 155.4, 146.4, 140.3, 137.9, 132.1, 129.5, 129.3, 126.2, 125.6, 113.8, 62.2, 60.9, 21.4, 13.2, 12.5. ESI-MS: calcd for $C_{18}H_{19}NO_3$ $[M+H]^+$: 298.36; found, 298.14.

4,9-dimethoxy-3-(p-tolyl)-5,6,7,8-tetrahydronaphtho[2,3-d]isoxazole (9746). White solid in 73.2% yield. ^{1}H-NMR (400 MHz, DMSO-d_6) δ 7.78 (d, J = 8.0 Hz, 2H), 7.39 (d, J = 7.9 Hz, 2H), 4.05 (s, 3H), 3.26 (s, 3H), 2.85- 2.68 (m, 4H), 2.42 (s, 3H), 1.82-1.65 (m, 4H). ^{13}C-NMR (100 MHz, DMSO-d_6) δ 157.3, 154.4, 146.0, 140.3, 137.2, 132.2, 129.6, 129.2, 126.7, 125.6, 113.7, 61.8, 60.4, 24.1, 23.3, 22.1, 22.1, 21.4. ESI-MS: calcd for $C_{20}H_{21}NO_3$ $[M+H]^+$: 324.47; found, 324.25.

5,6-dichloro-4,7-dimethoxy-3-(p-tolyl)benzo[d]isoxazole (9747). White solid in 8.7% yield. ^{1}H-NMR (400 MHz, Acetone-d_6) δ 7.83 (d, J = 8.2 Hz, 2H), 7.44 (d, J = 7.9 Hz, 2H), 4.26 (s, 3H), 3.51 (s, 3H), 2.46 (s, 3H). ^{13}C-NMR (100 MHz, Acetone-d_6) δ158.5, 156.1, 146.4, 141.6, 138.7, 130.1, 126.7, 125.5, 122.8, 116.9, 62.6, 61.6, 21.4. ESI-MS: calcd for $C_{16}H_{13}Cl_2NO_3$ $[M+H]^+$: 338.07; found, 338.03.

3-(4-fluorophenyl)benzo[d]isoxazole (9748). To a solution of 4-fluoro-N-hydroxybenzimidoyl chloride (1.2 mmol), anthranilic acid (2.3 mmol) and K_2CO_3 (1.7 mmol) in CH_3CN (15.0 mL) was added tert-butyl nitrite (2.3 mmol) at room temperature. The mixture was stirred at 115°C for 20 h. Then the mixture was evaporated to get the crude product. The compound was obtained and characterized after purification by silica gel column chromatography (ethyl acetate/petroleum ether). Yellow powder in 41.3% yield. ^{1}H-NMR (400 MHz, $CDCl_3$) δ 8.03-7.92 (m, 2H), 7.90 (d, J = 8.0 Hz, 1H), 7.60-7.64 (m, 2H), 7.43-7.37 (m, 1H), 7.24-7.29 (m， 2H). ^{13}C-NMR (100 MHz, $CDCl_3$) δ 165.2, 163.9, 162.7, 156.4, 130.0, 129.9, 129.8, 125.15, 125.12, 124.0, 121.9, 120.3, 116.4, 116.2, 110.2. ESI-MS: calcd for $C_{13}H_8FNO$ $[M+H]^+$: 214.51; found, 214.06.

N-(2-(4-((3-(4,9-dioxo-4,9-dihydronaphtho[2,3-d]isoxazol-3-yl)phenoxy)methyl)-1H-1,2,3-triazol-1-yl)ethyl)-5-((3aS,4S,6aR)-2-oxohexahydro-1H-thieno[3,4-d]imidazol-4-yl)pentanamide (9788). To a solution of **9787** (1.0 mmol) and N-(2-azidoethyl)-5-((3aS,4S,6aR)-2-oxohexahydro-1H-thieno[3,4-d]imidazol-4-yl)pentanamide (1.2 mmol) in N,N-dimethylformamide (15.0 mL) and water (10.0 mL) was added $CuSO_4$ (1.5 mmol) and sodium ascorbate (0.5 mmol) at room temperature. The mixture was stirred at 43°C for 10 h. Then the mixture was poured into brine (60 mL) and extracted with ethyl acetate (20 mL × 3). The combined organic layer was washed with brine (50 mL × 2) and dried over Na_2SO_4. After evaporation of the solvent in vacuo, the compound was obtained and characterized after purification by silica gel column

chromatography (ethyl acetate/petroleum ether). Yellow powder in 53.5% yield. ^{1}H-NMR (400 MHz, DMSO-d_6) δ 8.25-8.27 (m, 3H), 8.01 (s, 3H), 7.79-7.82 (m, 1H), 7.68 (d, J = 6.3 Hz, 1H), 7.56 (s, 1H), 7.33-7.36 (m, 1H), 6.40 (d, J = 27.3 Hz, 1H), 5.26 (s, 1H), 4.45 (s, 2H), 4.20-4.23 (m, 2H), 3.51 (s, 2H), 3.36 (s, 3H), 3.08 (s, 1H), 2.79 (s, 1H), 2.03 (d, J = 6.3 Hz, 2H), 1.56 (s, 1H), 1.45-1.47 (m, 3H), 1.23-1.26 (m, 3H). ^{13}C-NMR (100 MHz, DMSO-d_6) δ 179.1, 173.7, 173.0, 167.1, 165.1, 160.5, 158.5, 143.2, 135.7, 134.9, 134.1, 132.3, 130.5, 127.7, 127.6, 127.5, 127.0, 122.0, 119.4, 117.9, 116.1, 61.8, 61.4, 59.6, 55.8, 49.4, 47.3, 35.4, 28.5, 28.4, 25.6. ESI-MS: calcd for $C_{32}H_{31}N_7O_6S$ $[M+H]^+$: 642.46 found, 642.21.

N-(2-(4-((3-(5,6-dimethyl-4,7-dioxo-4,7-dihydrobenzo[d]isoxazol-3-yl)phenoxy)methyl)-1H-1,2,3-triazol-1-yl)ethyl)-5-((3aS,4S,6aR)-2-oxohexahydro-1H-thieno[3,4-d]imidazol-4-yl)pentanamide (9791). Brown powder in 30.5% yield. ^{1}H-NMR (400 MHz, DMSO-d_6) δ 7.50-7.52 (m, 1H), 7.42 (t, J = 14.8 Hz, 1H), 7.27 (t, J = 3.0 Hz, 1H), 7.15 (s, 1H), 6.98-7.11 (m, 1H), 6.43 (s, 1H), 5.61 (t, J = 10.2 Hz, 2H), 5.55 (s, 1H), 5.42 (s, 1H), 5.16 (s, 2H), 4.57-4.61 (m, 1H), 4.48 (dd, J = 18.5, 16.1 Hz, 1H), 3.71 (t, J = 10.2 Hz, 2H), 3.45-3.33 (m, 3H), 2.35 (dt, J = 15.2, 11.6 Hz, 1H), 2.25-2.18 (m, 1H), 2.17-2.07 (m, 2H), 1.87 (s, 6H), 1.78-1.62 (m, 2H), 1.32-1.16 (m, 2H). ^{13}C-NMR (100 MHz, DMSO-d_6) δ 184.5, 176.7, 174.8, 163.7, 161.8, 160.5, 159.2, 143.0, 142.2, 141.7, 131.2, 128.8, 122.8, 121.9, 116.6, 112.6, 109.0, 68.1, 60.9, 56.6, 55.2, 49.9, 38.0, 37.4, 36.9, 29.9, 26.6, 25.3, 12.7. ESI-MS: calcd for $C_{30}H_{33}N_7O_6S$ $[M+H]^+$: 622.71 found, 620.52.
